# A Dual-Modality Imaging Method Based on Polarimetry and Second Harmonic Generation for Characterization and Evaluation of Skin Tissue Structures

**DOI:** 10.3390/ijms24044206

**Published:** 2023-02-20

**Authors:** Liangyu Deng, Zhipeng Fan, Binguo Chen, Haoyu Zhai, Honghui He, Chao He, Yanan Sun, Yi Wang, Hui Ma

**Affiliations:** 1 Guangdong Research Center of Polarization Imaging and Measurement Engineering Technology, Shenzhen Key Laboratory for Minimal Invasive Medical Technologies, Institute of Biopharmaceutical and Health Engineering, Tsinghua Shenzhen International Graduate School, Tsinghua University, Shenzhen 518055, China; 2 Department of Biomedical Engineering, Tsinghua University, Beijing 100084, China; 3 Department of Engineering Science, University of Oxford, Parks Road, Oxford OX1 3PJ, UK; 4 Experimental Research Center, China Academy of Chinese Medical Sciences, Beijing 100700, China; 5 Department of Physics, Tsinghua University, Beijing 100084, China

**Keywords:** Mueller matrix polarimetry, second harmonic generation, microscopy, dual-modality imaging, skin tissue structures

## Abstract

The characterization and evaluation of skin tissue structures are crucial for dermatological applications. Recently, Mueller matrix polarimetry and second harmonic generation microscopy have been widely used in skin tissue imaging due to their unique advantages. However, the features of layered skin tissue structures are too complicated to use a single imaging modality for achieving a comprehensive evaluation. In this study, we propose a dual-modality imaging method combining Mueller matrix polarimetry and second harmonic generation microscopy for quantitative characterization of skin tissue structures. It is demonstrated that the dual-modality method can well divide the mouse tail skin tissue specimens’ images into three layers of stratum corneum, epidermis, and dermis. Then, to quantitatively analyze the structural features of different skin layers, the gray level co-occurrence matrix is adopted to provide various evaluating parameters after the image segmentations. Finally, to quantitatively measure the structural differences between damaged and normal skin areas, an index named Q-Health is defined based on cosine similarity and the gray-level co-occurrence matrix parameters of imaging results. The experiments confirm the effectiveness of the dual-modality imaging parameters for skin tissue structure discrimination and assessment. It shows the potential of the proposed method for dermatological practices and lays the foundation for further, in-depth evaluation of the health status of human skin.

## 1. Introduction

Skin diseases are prevalent and affect more than 30% of individuals worldwide [1,2,3,4,5,6]. During the development of skin diseases, structural changes in the different skin layers (stratum corneum, epidermis, dermis, and hypodermis) often occur [7,8,9,10]. The characterization of layered skin tissue structures can provide crucial information for various applications, ranging from transdermal drug delivery [11], cancer diagnosis [12], skin lesion detection [13], and wound healing and scarring evaluation [14]. Currently, the gold standard for diagnosing pathological skin diseases is the microscopic observation of histological tissue slices, which requires staining skin tissue sections with certain dyes and evaluation by experienced pathologists [15]. The lack of quantitative evaluation indices often results in significant interobserver and intraobserver variabilities. Thus, a quantitatively reliable method allowing for characterizing skin tissue structures would have a good application prospect in dermatological practices.

Mueller matrix polarimetry has recently gained popularity in biomedical research due to its high potential for detecting the microstructural and optical properties of label-free samples [16,17,18,19,20,21]. Studies have demonstrated that Mueller matrix-derived parameters are sensitive to different structures and have significant application prospects in the pathological diagnosis of various abnormal tissues, such as skin cancer [22], breast cancer [23,24], liver cirrhosis and cancer [25,26], thyroid cancer [27], colon cancer [28,29,30], cervical cancer [31,32,33], and lung cancer [34]. Mueller matrix polarimetry can also be used to visualize brain white matter fiber tracts [35], explain the combined effects of scattering and absorption changes in cancer growth [36], and assess β-amyloidosis in Alzheimer’s disease [37]. Specifically, for skin tissue, its highly anisotropic nature induced by different ultra-structural components such as collagen, elastin matrix, and fibers makes it a particularly suitable investigation site for Mueller matrix polarimetry [38].

On the other hand, with the advantages of high imaging resolution, reasonable penetration depth, and low phototoxicity, second harmonic generation (SHG) microscopy has become a prevalent tool for biomedical detection and disease diagnosis [39,40,41,42]. For skin tissue imaging, abundant collagen fibers distributed inside the dermis often produce high second-harmonic signals [43,44]. Thus, the thickness, density, and orientation of skin collagen fibers can be quantitatively evaluated by SHG imaging [45,46,47]. Moreover, it has been demonstrated that polarization-sensitive SHG signals can effectively extend the SHG imaging potential for extracting more detailed skin tissue structural information [48].

Considering that the structural features of skin tissue are often too complicated to use a single imaging modality for a comprehensive evaluation, it is of great significance to exploit multimodal imaging methods suitable for skin tissue characterization. In this study, by combining the advantages of Mueller matrix polarimetry and SHG microscopy, a dual-modality imaging method is proposed. This method can clearly identify the characteristic layered structures of mouse tail skin tissue and provide a tool for the following evaluation of the stratum corneum, epidermis, and dermis. We also adopt the gray level co-occurrence matrix (GLCM) to quantitatively analyze the structural features of different skin tissue layers after the image segmentations. Finally, to quantitatively measure the structural differences between damaged and normal skin model tissue areas, an index named Q-Health is defined based on cosine similarity and GLCM parameters. The experimental results confirm the effectiveness of the dual-modality imaging parameters for skin tissue structure discrimination and assessment. It shows the potential of the proposed method for dermatological diagnosis and lays the foundation for the evaluation of the health status of human skin.

## 2. Results and Discussions

### 2.1. Microscopic Imaging Results of Skin Tissue Samples

**Figure 1:**
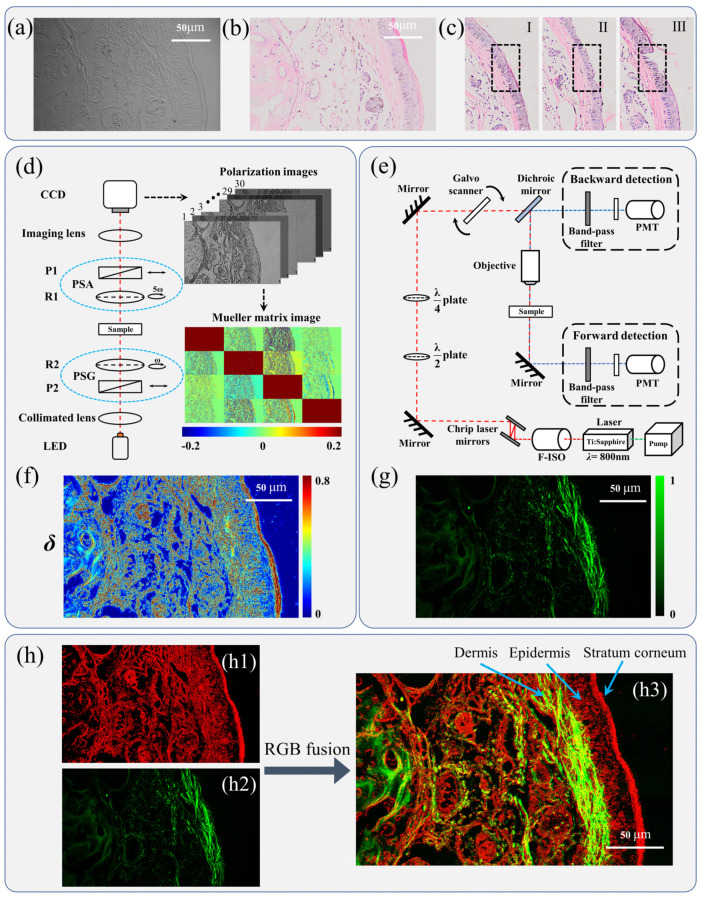
Schematics of skin tissue samples and experimental setups: microscopic images of mouse tail skin tissues for (**a**) the unstained slice, (**b**) the corresponding H&E-stained slice using a 20×/0.75 NA objective (UPlanSApo, Olympus, Tokyo, Japan); (**c**) H&E-stained tissue sections with damage areas in different layers indicated by dashed boxes, I: stratum corneum layer, defined as having a depth of less than 40 μm damage with partial loss of the stratum corneum layer, II: stratum corneum and epidermis layers, defined as having a depth between 40 and 140 μm damage, with complete destruction of the stratum corneum layer and partial destruction of the epidermis layer, III: stratum corneum, epidermis, and dermis layers, defined as having a depth larger than 140 μm damage to the skin dermis layer; (**d**) schematic of the Mueller matrix microscope: LED (XLamp XP-E, 3.5 W, 633 nm, Δλ = 20 nm, Cree Inc., Durham, NC, USA), P: polarizer (extinction ratio 1000:1, Daheng Optics, Beijing, China), R: quarter-wave plate (Daheng Optics, Beijing, China), PSG: polarization state generator, PSA: polarization state analyzer, CCD (74-0107A, 12-bit, QImaging, Surrey, BC, Canada); (**e**) schematic of the SHG microscope, PMT: photomultiplier tube, F-ISO: Faraday isolator; (**f**) MMPD-*δ* image of the mouse skin tissue slice; (**g**) SHG image of the same mouse skin tissue area; (**h**) RGB fusion process, (**h1**): MMPD-*δ* image in R channel, (**h2**): SHG image in G channel, (**h3**): dual-modality fusion result.

Figure 1a shows the microscopic unpolarized intensity image of a 4-μm-thick, dewaxed, unstained transverse tissue section of normal mouse tail skin using a 20× objective lens. Figure 1b shows the image of the corresponding H&E-stained slice of the same area. It can be observed that the unpolarized intensity image of an unstained tissue slice roughly shows the outlines and mixed undulating gully structures, making it challenging to separate the layers of skin tissue. On the other hand, the image of the H&E-stained slice clearly shows each layer of the skin, including the outermost red stratum corneum, the middle lavender epidermis, and the inner pale pink dermis. Figure 1c shows the images of H&E-stained slices of damaged skin models: I is the stratum corneum layer damaged model; II is the stratum corneum and epidermis layers damaged model; III is the stratum corneum, epidermis, and dermis layers damaged model. The damaged areas are marked by the dashed rectangles. For stratification and quantitative assessment, both the microscopic Mueller matrix and SHG images of the samples were acquired.

From the Mueller matrix image of the sample shown in Figure 1d, we can see that there are apparent imaging contrasts in the elements *M*_24_, *M*_34_, *M*_42_, and *M*_43_, indicating that the skin tissue has a strong linear birefringence effect. Therefore, we further decomposed the Mueller matrix to get the Mueller matrix polar decomposition (MMPD)-*δ* image, as shown in Figure 1f. It can be intuitively seen from the MMPD-*δ* image that the outermost stratum corneum layer of the skin tissue has a concentrated and strong signal, while the signals of other layers are relatively weak and scattered. This phenomenon is probably due to the strong birefringence effect induced by the neat and dense protein structures in the stratum corneum that form the skin barrier. Thus, the MMPD-*δ* image can be used to distinguish the stratum corneum from other skin layers.

From the SHG image of the same sample area shown in Figure 1g, we can observe a randomly arranged network structure with strong SHG signals, resulting from the non-centrosymmetric and long-range ordered collagen fibers in skin. Specifically, collagen fibers are the main components in the dermis, accounting for 95–98% of the total fiber mass, while there are no collagen fibers in the stratum corneum and epidermis [49]. It means SHG imaging can be used to identify and characterize the dermal layer of the skin.

According to the above results, the information provided by dual-modality imaging of the Mueller matrix and SHG can be used to distinguish between different skin layers. As shown in Figure 1h, we fused the two imaging modalities’ results via the RGB channels [38]. Here the MMPD-*δ* and SHG images were input to the R and G channels, respectively, while the B channel was set to be 0. In the fusion process, the feature point matching method was adopted to deal with the pixels registration problem between the MMPD-*δ* and SHG images foremost, since the sizes of these two modalities images do not match. Based on the approximate local transformation of feature points [50], this method registers images by mathematically transforming a series of corresponding feature points on the MMPD-*δ* and SHG images. Figure 1(h3) shows the images fusion result, in which the left region or the dermis layer appears yellow-green because of the superimposition between red MMPD-*δ* and green SHG images; while the outermost dense red region shows the stratum corneum layer, and the middle between the two regions is the epidermis layer. The results shown in Figure 1 demonstrated that the stratum corneum, epidermis, and dermal layers can be effectively discriminated by using the dual-modality imaging method.

### 2.2. Skin Layers Segmentation Results

**Figure 2:**
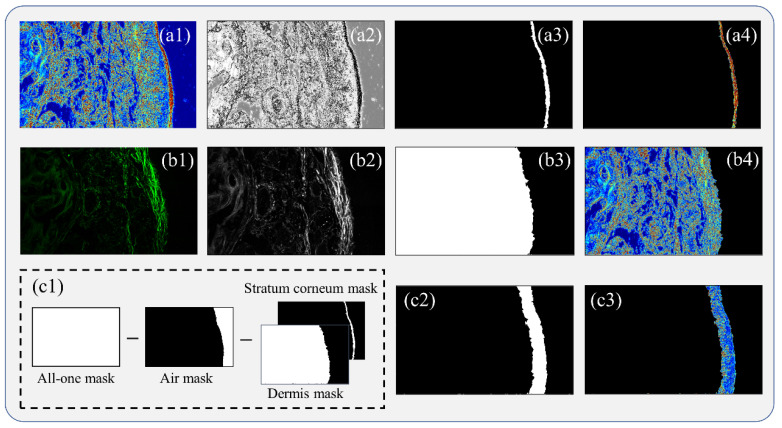
Skin layer segmentation process based on dual-modality Mueller matrix and SHG imaging: (**a1**–**a4**) stratum corneum layer segmentation process: (**a1**) MMPD-*δ* image, (**a2**) gray-scale image converted from the MMPD-*δ* image, (**a3**) stratum corneum mask, (**a4**) segmented stratum corneum layer; (**b1**–**b4**) dermis layer segmentation process: (**b1**) SHG image, (**b2**) gray-scale image converted from the SHG image, (**b3**) dermis mask, (**b4**) segmented dermis layer; (**c1**–**c3**) epidermis layer segmentation process: (**c1**) all-one mask, air mask, stratum corneum mask, and dermis mask, (**c2**) epidermis mask, (**c3**) segmented epidermis layer.

As shown in Figure 2, we then segmented the three layers of skin tissue using the dual-modality image. The layer segmentation is the basis for the following quantitative damage assessment. Figure 2(a1–a4) show the segmentation process of the stratum corneum, which was acquired by multiplying the original MMPD-*δ* image and the corresponding mask obtained by the regional growth algorithm. It is worth noting that the algorithm was applied to the gray-scale image converted from the MMPD-*δ* imageFigure 2(b1–b4) show the segmentation process of the dermis. The SHG image clearly identifies the dermal abundance of non-neutral symmetric collagen fibers. There is no SHG signal in the epidermis and stratum corneum layers without collagen fiber. After the registration of the SHG and MMPD-*δ* images, the dermis layer was acquired by multiplying the original MMPD-*δ* image and the corresponding mask obtained by morphological optimization methods such as median filtering, dilation, and filling. Figure 2(c1–c3) show the segmentation process of the epidermis, which was acquired by multiplying the original MMPD-*δ* image and the corresponding mask obtained by subtracting the air mask from the regional growth algorithm, the stratum corneum mask (shown in Figure 2(a3)), and the dermis mask (shown in Figure 2(b3)) from an all-one mask with the same size as the MMPD-*δ* image in turn. All 15 skin tissue images were sequentially segmented according to the process indicated in Figure 2.

### 2.3. Quantitative Damage Assessment of Different Skin Layers

**Figure 3:**
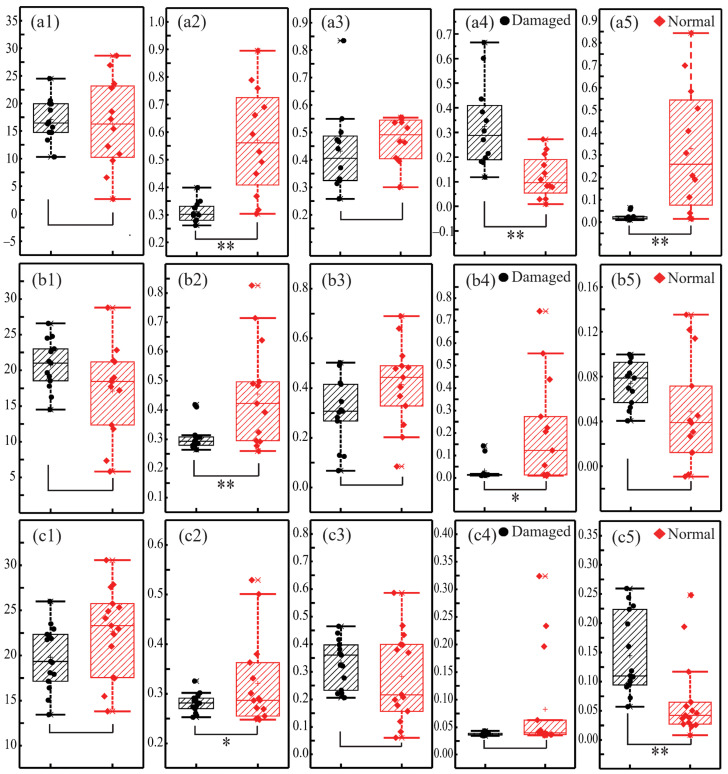
Quantitative damage assessment results of the damaged (black dots) and normal (red diamonds) regions: (**a1**–**a5**) distributions of the parameters contrast, homogeneity, correlation, energy, and mean for the stratum corneum layer; (**b1**–**b5**) distributions of the parameters contrast, homogeneity, correlation, energy, and mean for the epidermis layer; and (**c1**–**c5**) distributions of the parameters contrast, homogeneity, correlation, energy, and mean for the dermis layer. No “*” symbol means *p* > 0.5, or not significant; “*” means *p* < 0.05, or significant; “**” means *p* < 0.01, or very significant.

After the segmentation, damaged regions in each skin layer can be recognized, and the damage degree evaluation is of further interest. For quantification, we calculated the GLCM and first-order statistical parameters, namely contrast, homogeneity, correlation, energy, and mean of the damaged areas. Meanwhile, the same group of parameters for normal skin regions were also calculated for comparison. As an unbiased selection, each parameter was obtained by taking the average of five regions selected by experienced pathologists. The quantitative assessment results of the damaged and normal regions are shown in Figure 3, where the black dots and red diamonds represent the damaged and normal areas, respectively. The first row (Figure 3(a1–a5)), the second row (Figure 3(b1–b5)), and the third row (Figure 3(c1–c5)) show the results of the stratum corneum, epidermis, and dermis layers in turn. The first column through the fifth column, in turn, represent the values of contrast, homogeneity, correlation, energy, and mean. In addition, the data of damaged and normal areas from the same layer in each sub-figure were compared by using the *t*-test. No “*” symbol means *p* > 0.5, or not significant; “*” means *p* < 0.05, or significant; “**” means *p* < 0.01, or very significant. It should be noted that samples with invalid values were excluded from the calculation process. Finally, there were 12 stratum corneum samples, 13 epidermis samples, and 15 dermal samples for analysis. First, we can observe from Figure 3(a1–c1) that the T-test analysis of the contrast shows no significant distinctions between damaged and normal areas for all three skin layers, which confirms the fixed-point damage has a limited effect on the texture depth of each skin layer. The value distribution of the contrast parameter indicates that the damage does not significantly change the difference between the maximum and minimum values of the linear retardation of the skin sample. Second, it can be noticed from Figure 3(a2–c2) that the T-test values of homogeneity for all three layers show significant distinctions, indicating that the local distribution of linear retardation resulting from fibers changes very frequently, which is consistent with the damage-induced irregular structural distribution. Third, as illustrated in Figure 3(a3–c3), the correlation values of the normal regions are larger than those of the damaged regions in both the stratum corneum and epidermis layers, while the opposite trend happens for the dermis layer. It proves that, compared to the dermis, the structures of the stratum corneum and epidermis are neater. Though there is no significant distinction, the correlation can also be used to describe the consistency of the texture declining after damage. Fourth, as shown in Figure 3(a4–c4), the energy values change significantly after the stratum corneum and epidermis layers were damaged, which indicates that the texture uniformity of the MMPD-*δ* images of these two layers change prominently. That may be related to the disorder of the arrangement or fluctuation angle of microstructures. However, for the dermis layer, the energy values are relatively small for both normal and damaged areas. It verifies that the dermal structure is looser and more disordered compared to those of the other two layers. Last, Figure 3(a5–c5) show the mean values to reflect the sample population information. Obviously, the mean values of the stratum corneum and dermal layers change significantly after the damage, but not in the epidermis. It shows that a change occurred in the fundamental frequency signal of MMPD-*δ* images after the stratum corneum and epidermis layers were damaged to be more dispersal, while the change in the epidermis is not significant because of the fundamental frequency signal of the MMPD-*δ* images here is generally low. Combining the mean value with the GLCM statistic parameters, a quantitative and comprehensive evaluation of the skin structures can be achieved. 

In summary, the parameters listed in Figure 3 can provide quantitative structural information about the skin sample. Specifically, for the stratum corneum damage models, significant differences exist in the three parameters of homogeneity, energy, and mean. While for the epidermis damage cases, the two parameters of homogeneity and energy show statistical differences. As for the dermal damage cases, the mean is statistically different. The results demonstrate that combining the five parameters into a new index for comprehensive skin damage evaluation is promising.

### 2.4. Q-Health Index Analysis

**Figure 4:**
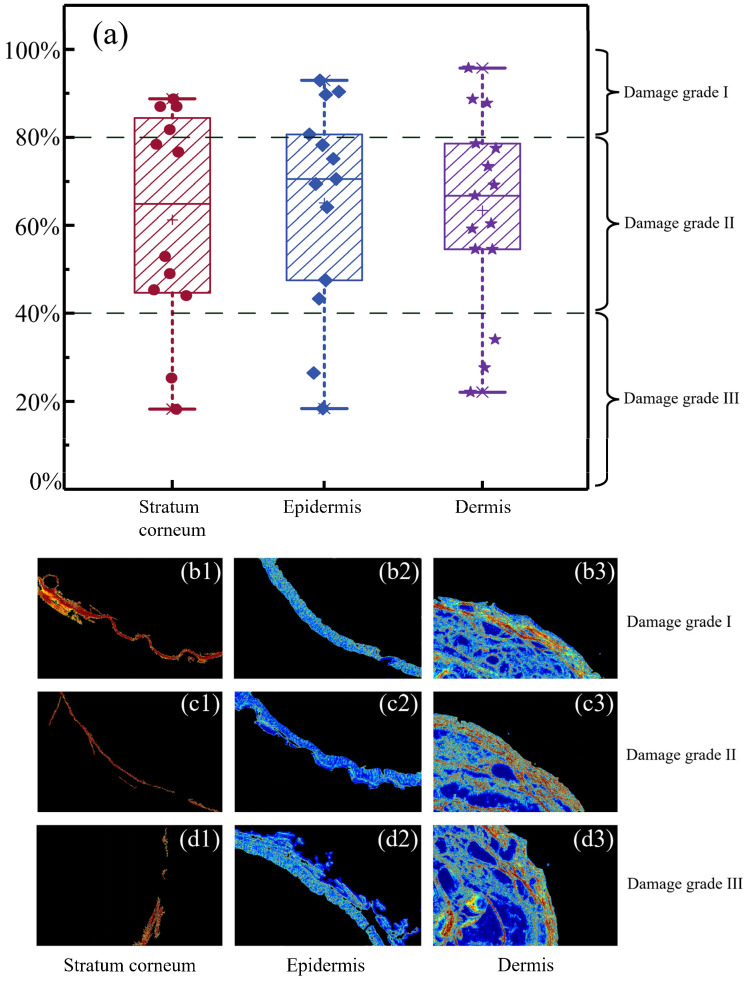
Q-Health index analyzing results: (**a**) Q-Health index distributions of all samples, the red dots represent the Q-Health values of the damaged stratum corneum samples, the blue diamonds represent the Q-health values of the damaged stratum corneum samples, the purple pentagrams represent the Q-health values of the damaged dermis samples, the “×” symbols represent the maximum and minimum Q-health values of each group, and the “+ ” symbols represent the average Q-health values of each group; (**b1**–**b3**) stratum corneum, epidermis, and dermis samples of damage grade I; (**c1**–**c3**) stratum corneum, epidermis, and dermis samples of damage grade II; (**d1**–**d3**) stratum corneum, epidermis, and dermis samples of damage grade III.

The Q-Health index can be calculated according to Equation (5), where the feature vectors of damaged and normal skin tissue samples were constructed via the five parameters as shown in Figure 3. Figure 4a shows the Q-Health index analyzing results for all the samples. For confirmation, here are the damage grades of specimens assessed by the pathologists from the Chinese Academy of Chinese Medical Sciences.

As shown in Figure 4a, according to the pathological evaluation results for the stratum corneum, epidermis, and dermis skin layers, the Q-Health index distributed between 80 and 100% can be regarded as damage grade I, that distributed between 40 and 80% can be regarded as damage grade II, and that distributed between 0 and 40% as damage grade III. As the detailed examples show, Figure 4(b1–b3) show the stratum corneum, epidermis, and dermis layers of damage grade I, respectively. Their Q-Health index values are distributed in the range of 80–100%, indicating that the deviation of the feature vectors between the normal and damaged regions is slight. It reflects the complete appearance of the tissue in grade I, and no fracture or ulceration occurs. Figure 4(c1–c3) and Figure 4(d1–d3) are the stratum corneum, epidermis, and dermal layers of damage grades II and III, respectively. With the decrease of the Q-Health index values, the damaged regions gradually appear faulty, where the tissue structures become loose. We can notice that there are large areas of cavities, and the textures of the images have been considerably changed. Table 1 shows the Q-Health index values of the samples in Figure 4. It can be observed from Figure 4 and Table 1 that the Q-Health index has the potential for quantitative and automatic assessment of skin tissue damage grade in different layers.

It is worth mentioning that there are also several other techniques available for the characterization and quantitative evaluation of layered skin tissue structures, each with its own strengths and limitations. Compared to histology [51] and confocal microscopy [52], the proposed dual-modality imaging method can provide real-time results of skin tissue structure and composition. Compared to optical coherence tomography [53] and ultrasound imaging [54], the proposed method can provide a higher imaging resolution of skin tissue structure to distinguish different types of fibers. Additionally, compared to Raman spectroscopy, the proposed dual-modality imaging method can provide more detailed information about the orientation and density of collagen fibers, which are crucial for understanding the mechanical properties and function of the skin tissue [55].

The developed dual-modality imaging method has been proven to be a valuable tool for characterizing and quantitatively evaluating skin tissue due to its advantages, such as label-freeness, high sensitivity, and specificity. As a result, it holds the potential to be applied to a variety of tissues. For instance, it may be useful in the examination of extracellular matrix in cardiovascular [56] and respiratory tissues [57] or in characterizing connective tissues in musculoskeletal systems [58]. Nevertheless, additional research is needed to assess the ability of this technique for the characterization of other tissues and to develop approaches for optimizing the imaging method for each specific tissue.

## 3. Materials and Methods

### 3.1. Mouse Tail Skin Tissue

As shown in Figure 1a, the tissue samples used in this study are 4-μm-thick, dewaxed, unstained transverse slice sections of mouse tail skin, provided and prepared by the Experimental Research Center, China Academy of Chinese Medical Sciences. Before measurement, the stratum corneum, epidermis, and dermis of the tissue sections were identified and fixed-point damaged by experienced pathologists. Each layer contains both damaged and normal areas. In total, we obtained 15 samples with normal and damaged dermis, 15 samples with normal and damaged epidermis, and 15 samples with normal and damaged stratum corneum. After acquiring their Mueller matrix microscopic and SHG images, the tissue sections were stained with hematoxylin and eosin (H&E) for the following pathological observations: The stained normal mouse tail skin tissue structure and damaged areas in various skin layers are shown in Figure 1b,c. The study was approved by the Ethics Committee of the Tsinghua Shenzhen International Graduate School.

### 3.2. Mueller Matrix Microscope

The schematic of the Mueller matrix microscope used in this study is shown in Figure 1d. By adding the polarization state generator and analyzer (PSG and PSA) modules to the optical path of a transmitted light microscope (L2050, Liss Optical Instrument Factory, Guangzhou, China), the setup can measure the Mueller matrix of a sample based on the dual-rotating retarder method [59,60,61]. During each measurement, two fixed linear polarizers (P1, P2, extinction ratio 1000:1, Daheng Optics, Beijing, China) and two rotatable quarter-wave plates (R1, R2, Daheng Optics, Beijing, China) are combined to achieve different polarization state modulations. Specifically, the quarter-wave plates are driven by the servo motor drivers (PRM1Z8E, Thorlabs Inc., Newton, NJ, USA) to rotate 30 times. R1 is rotated 6 deg, and R2 is rotated 30 deg each time. After the rotation, thirty polarized light intensity images are collected by the gray-scale CCD (74-0107A, 12-bit, QImaging, Surrey, BC, Canada). Then, the Mueller matrix of the sample can be calculated based on Fourier analysis using the coefficients *α_n_* and *β_n_* shown in Equation (1)

(1) $$I=\alpha_{0}+\sum_{n=1}^{12}\left(\alpha_{n}\cos2n\theta +\beta_{n}\sin2n\theta \right)$$

where *I* is the light intensity image collected by the CCD each time, and the Fourier coefficients *α_n_* and *β_n_* are the functions of the 16 Mueller matrix elements, each of which can be calculated by inverse operation. *θ* is the angle of each rotation of the quarter-wave plate R1.

More details of the dual-rotating retarder method and the calculation process of the Mueller matrix can be found in [62,63]. Before measurements, the microscope was calibrated by measuring standard samples such as air, polarizers, and quarter-wave plates along different axis directions [64]. The results showed that the maximum error of the measured Mueller matrix element is about 1%.

### 3.3. Mueller Matrix Polar Decomposition

Mueller matrix polarimetry has been proven to be a powerful tool for probing the microstructures of biological tissues [16]. However, it is inconvenient to directly use a single Mueller matrix element for structure detection and evaluation since it lacks a clear association with certain microstructures [21,65,66]. To address this issue, several Mueller matrix analyzing methods have been proposed over the years, including the Mueller matrix polar decomposition (MMPD) technique, which is widely used in biomedical studies and clinical applications. The MMPD method derives parameters from the Mueller matrix that are more relevant to the microstructural features of interest [67]. Specifically, the MMPD decomposes the interaction between light and medium into three main processes of polarization properties, namely diattenuation (*D*), retardation (*R*), and depolarization (Δ), as shown in Equation (2). Further, through the decomposition process, we can obtain a group of polarization parameters, among which the MMPD-*D*, MMPD-*δ*, and MMPD-Δ corresponding to dichroism, linear retardation, and depolarization are mostly used in biomedical trials [68,69]. The detailed polar decomposition process is shown in Equation (2)

(2) $$M=M_{\Delta }M_{R}M_{D}\;D=\sqrt{M_{12}^{2}+M_{13}^{2}+M_{14}^{2}}\;\delta =\arccos(\sqrt{{\left(M_{R22}+M_{R33}\right)}^{2}+{\left(M_{R32}-M_{R23}\right)}^{2}}-1)\;\Delta =1-\frac{1}{3}\left|tr(M_{\Delta })\right|$$

where *M* is the measured Mueller matrix, *M_ij_* is the element of *M*; *M*_∆_, *M_R_* and *M_D_* are the sub-matrices of depolarization, retardation and diattenuation, respectively, *M_Rij_* is the element of *M_R_; D* is the diattenuation, *δ* is the linear retardation, Δ is the depolarization.

Skin tissues are thin, heterogeneously layered media with significant linear retardation distribution due to different cell types and tissue densities [38]. Therefore, in this study, we employ the MMPD parameter *δ* reflecting linear retardation for the evaluation of mouse skin tissue samples.

### 3.4. Second Harmonic Generation Microscope

The schematic of the SHG microscope (LMS 710, Zeiss, Jane, Germany) is shown in Figure 1e. The confocal microscope is equipped with a Ti:Sapphire Chameleon multiphoton tunable laser (Coherent, Santa Clara, CA, USA) at 800 nm. For the SHG imaging, a dichroic mirror, a custom filter set (BP: 414/46, DC: 495, and BP: 525/50), and a 20× water immersion objective are used.

### 3.5. Image Segmentation Algorithms

For quantitative evaluation of the mouse tail skin tissue samples, we segmented the skin layers based on both the MMPD-*δ* and SHG images. Here the widely acknowledged three-layered model is adopted to divide the skin structure into the stratum corneum, epidermis, and dermis, which show differences in the optical anisotropy [70]. To accurately segment the three layers, we employed the regional growth algorithm, which is often used in recognition tasks such as remote sensing [71] and disease diagnosis with high simplicity and efficiency [72]. The regional growth algorithm collects similar pixels to form regions as follows: First, multiple initial points of the segmented region are selected as seeds; second, a similarity evaluation is performed to determine whether or not to grow; and last, the growth is stopped until a certain threshold is reached. The accuracy of segmentation depends significantly on the threshold selection [73]. Thus, to ensure the adaptability of the algorithm and the accuracy of segmentation, we used the maximum inter-class variance method to find the best threshold in each image. The maximum inter-class variance method proposed by Otsu in 1978 [74] is an adaptive algorithm that calculates the inter-class variance based on the grayscale characteristics of the image and finds the grayscale value corresponding to the maximum inter-class variance as the optimal threshold. The schematic of the skin layer segmentation process based on dual-modality Mueller matrix and SHG imaging in this study is shown in Figure 2.

### 3.6. Image Texture Analysis

To further quantitatively assess the damage degree of each skin layer, we performed texture analysis on MMPD-*δ* images using the GLCM method [75]. The GLCM is a vital method to characterize texture differences based on gray-scale spatial distribution and has shown great potential for the detection and quantitative staging of abnormal tissues [76]. Here, the GLCM parameters contrast, correlation, homogeneity, and energy shown in Equation (3) are chosen to analyze the texture features of the MMPD-*δ* images. Among them, (a) contrast represents the depth of the texture. The smaller the contrast, the less difference there is between the gray levels, and the shallower the image texture grooves; (b) the homogeneity measures the local change of the image texture. The smaller the homogeneity, the more uneven the local area, and the more frequent the changes between different texture regions, (c) the correlation shows the consistency of image texture. The smaller the correlation, the less similar the pixels are in the row or column direction, and the greater the difference between the pixels. (d) The energy reflects the uniformity of the gray-scale distribution of the image. The smaller the energy, the more uneven the distribution of an image texture.

In this study, the region of interest (ROI) was selected under experienced pathologists’ guidance, including the damaged and normal regions. The size of the ROI was 20 pixels × 20 pixels. The GLCM was calculated using MATLAB (graycomatrix function [77]), and four correlation statistics were derived from the obtained GLCM through MATLAB (graycoprops function [78]). The gray value range of the MMPD-*δ* images is normalized to [0, 255], the gray level *N_g_* is set to 64, and the inter-pixel displacement *d* is 1. Each derived correlation statistic is the average of the features in the four directions (0°, 45°, 90°, and 135°).

(3) $$p_{x}(i)=\sum_{j=1}^{N_{g}}p(i,j),p_{y}(j)=\sum_{i=1}^{N_{g}}p(i,j)\;Contrast=\sum_{n=0}^{N_{g}-1}n^{2}\{\sum_{i=1}^{N_{g}}\sum_{j=1}^{N_{g}}p(i,j)||i-j|=n\}\;Homogeneity=\sum_{i}\sum_{j}\frac{1}{{1+(i-j)}^{2}}p(i,j)\;Correlation=\frac{\sum_{i}\sum_{j}(ij)p(i,j)-\mu_{x}\mu_{y}}{\sigma_{x}\sigma_{y}}\;Energy={\sum_{i}\sum_{j}p(i,j)}^{2}$$

In Equation (3), *N_g_* is the quantized gray level, *p*(*i*, *j*) is the relative probability that two gray levels of *i* and *j* appear on the image by *d* pixels displaced in a particular direction, *µ_x_*, *µ_y_*, *σ_x_*, and *σ_y_* are the mean and standard deviation of *p_x_* and *p_y_.*

### 3.7. Q-Health Index

To seek a comprehensive index for characterizing and evaluating the damage degree of skin tissue layers, we propose the Q-Health, a custom index based on cosine similarity and GLCM parameters. Cosine similarity converts the similarity measure into an angle between two vectors, which has many applications because of its simplicity and practicality, such as document information retrieval [79] and face recognition [80]. The construction of the Q-Health index includes two steps. First, a 5-dimensional feature vector is constructed by synthesizing the GLCM texture parameters contrast, correlation, homogeneity, energy, and the first-order statistical feature mean as shown in Equation (4)

(4) $$Mean=\sum_{i}z_{i}p(z_{i})$$

where *p*(*z_i_*) is the proportion of pixels with the value of *z_i_* to the total number of pixels. Second, the Q-Health index is obtained by calculating the cosine similarity between the feature vectors of the damaged and normal skin regions, as shown in Equation (5)

(5) $$Q-Health=\frac{A\cdot B}{\left\|A\right\|\left\|B\right\|}=\frac{\sum_{i=1}^{n}A_{i}\times B_{i}}{\sqrt{\sum_{i=1}^{n}{\left(A_{i}\right)}^{2}}\times \sqrt{\sum_{i=1}^{n}{\left(B_{i}\right)}^{2}}}$$

where *A* and *B* are the feature vectors of the damaged skin region and the normal skin region, *n* is the dimension of the eigenvectors, and here is 5. The method is inspired by the ability of cosine similarity to assess the deviation between two vectors. To measure the damage degree, we used the feature vector of the normal skin region as a benchmark and then calculated the deviation between the benchmark and the feature vector of the damaged region of the corresponding skin layer. The Q-Health index has a positive value distributed from 0 to 1 because the feature vectors in this study are positive. The larger value of the Q-Health index means a smaller deviation.

## 4. Conclusions

In this study, we proposed a dual-modality imaging method based on Mueller matrix polarimetry and second harmonic generation to realize the characterization and quantitative evaluation of layered skin tissue structures. The imaging results of mouse skin tissue slices showed that the stratum corneum layer of the skin tissue has a concentrated and strong linear retardance signal induced by the neat and dense protein structures that form the skin barrier. As a result, the MMPD-*δ* image can be used to distinguish the stratum corneum from other skin layers. Meanwhile, SHG imaging can be used to identify and characterize the dermal layer of the skin, which is abundant in collagen fibers and generates strong SHG signals. We demonstrated that the stratum corneum, epidermis, and dermis layers of skin tissue specimens can be effectively segmented using dual-modality images. Then, the GLCM method was carried out to analyze the texture features of different skin layers after the segmentations. The results showed significant differences in the MMPD-*δ* parameter images among different skin layers and areas, indicating that the GLCM method can provide the metrics for skin tissue structures assessment. Finally, to quantitatively evaluate the structural differences between damaged and normal skin tissues, we proposed the Q-Health index based on the cosine similarity to measure the texture feature vector deviation between the damaged and normal areas. The experimental results confirmed that, the texture features of the MMPD-*δ* parameter images could be used for the accurate characterization and evaluation of skin tissue structures. It shows the potential of the proposed method for dermatological diagnosis and lays the foundation for the evaluation of the health status of human skin.

## Figures and Tables

**Table 1:** Q-Health index values of the samples shown in Figure 4(b1–b3, c1–c3, and d1–d3).

	Damage Grade I	Damage Grade II	Damage Grade III
Stratum corneum	88.8%	45.4%	18.3%
Epidermis	95.7%	47.5%	18.3%
Dermis	88.6%	60.1%	27.6%

## Data Availability

All data are available from the authors upon request.
